# Acute Generalized Exanthematous Pustulosis With Negative Patch Test and Drug-Induced Lymphocyte Stimulation Test (DLST) Results: A Case Report

**DOI:** 10.7759/cureus.82812

**Published:** 2025-04-22

**Authors:** Yoshihito Mima, Tsutomu Ohtsuka

**Affiliations:** 1 Department of Dermatology, Tokyo Metropolitan Police Hospital, Tokyo, JPN; 2 Department of Dermatology, International University of Health and Welfare, Tochigi, JPN

**Keywords:** acute generalized exanthematous pustulosis, drug eruption, drug-induced lymphocyte stimulation test, false negative, patch test

## Abstract

Acute generalized exanthematous pustulosis (AGEP) is a rapid-onset skin disorder characterized by edematous erythema, mainly affecting intertriginous areas and the face, along with sterile, non-follicular pustules. It is most often drug-induced, commonly triggered by antibiotics, anticancer agents, or hydroxychloroquine. Symptoms typically appear within a few days of drug exposure. Histologically, AGEP features spongiform pustules in or beneath the stratum corneum, papillary dermal edema, and perivascular neutrophilic infiltration. Patch testing and drug-induced lymphocyte stimulation test (DLST) are used to identify the causative agent, although both may yield false-negative results. We report a case of AGEP occurring two days after administration of paclitaxel, oral iron, and vonoprazan. All three drugs were discontinued, and the patient responded well to topical corticosteroids and oral prednisolone. Patch tests for all agents were negative. However, based on previous reports of severe vonoprazan-induced drug eruptions and the lack of evidence linking paclitaxel or oral iron to AGEP, vonoprazan was considered the most likely culprit. Since the patient wished to continue cancer treatment, paclitaxel and oral iron were cautiously reintroduced with informed consent and close monitoring. No recurrence occurred, further supporting vonoprazan as the causative agent. This case highlights the limitations of patch testing and DLST in AGEP diagnosis and emphasizes the importance of clinical context and therapeutic priorities in evaluating drug causality. Further studies are needed to standardize AGEP testing protocols and improve diagnostic accuracy.

## Introduction

Acute generalized exanthematous pustulosis (AGEP) is an acute-onset dermatological disorder characterized by edematous erythema, predominantly affecting intertriginous areas and the face. Numerous sterile, non-follicular pustules typically appear on the erythematous background. Patients often report a burning sensation or pruritus, and many develop fever exceeding 38°C along with leukocytosis, occasionally accompanied by mild eosinophilia [1]. Most cases of AGEP are drug-induced, with antibiotics, anticancer agents, and hydroxychloroquine being the most frequently implicated agents. Symptoms usually manifest within a few days of drug administration [2]. In recent years, associations between AGEP and viral infections, such as Epstein-Barr virus, parvovirus B19, cytomegalovirus, and herpesviruses, have also been reported, and cases following COVID-19 vaccination have been documented [2-5]. The pathogenesis of AGEP involves multiple immune pathways. Drug-activated CD4+ and CD8+ T cells induce keratinocyte apoptosis and tissue damage, leading to blister formation. Additionally, the secretion of interleukin (IL)-8 and IL-36 promotes neutrophil infiltration, contributing to pustule formation and peripheral neutrophilia [6]. Clinically, the pustules in AGEP typically resolve spontaneously within four to 10 days, often followed by desquamation and post-inflammatory hyperpigmentation. The prognosis is generally favorable; however, in elderly or immunocompromised patients, high fever and secondary bacterial infections may lead to more severe outcomes [1]. Histopathologically, AGEP is characterized by subcorneal or intraepidermal spongiform pustules, marked papillary dermal edema, and perivascular infiltration of neutrophils [7,8]. While perivascular neutrophilic infiltration and keratinocyte necrosis may suggest vasculitis, psoriasiform epidermal hyperplasia, typical of pustular psoriasis, is usually absent [7,8]. Diagnosis is based on clinical and histopathological findings, with the European Severe Cutaneous Adverse Reactions (EuroSCAR) scoring system serving as a diagnostic aid [9]. This system evaluates features such as pustule morphology, distribution of erythema, desquamation, rapid onset, changes in fever and leukocyte count, mucosal involvement, and histological findings. A score of 8 or higher confirms the diagnosis of AGEP [9]. Differential diagnoses include drug-induced hypersensitivity syndrome (DIHS)/drug reaction with eosinophilia and systemic symptoms (DRESS), toxic epidermal necrolysis (TEN), subcorneal pustular dermatosis, generalized pustular psoriasis, bacterial folliculitis, Kaposi varicelliform eruption, Sweet’s syndrome, and Behçet’s disease [1]. The first-line treatment is the discontinuation of the causative agent and the application of topical corticosteroids. In severe cases, oral prednisolone is recommended, along with symptomatic therapy using antipyretics and antihistamines. For cases unresponsive to systemic corticosteroids, cyclosporine may be considered as an alternative [1,2]. To identify the causative drug, patch testing and drug-induced lymphocyte stimulation test (DLST) may be employed; however, both tests can yield false-negative results [10,11]. Herein, given the rarity of AGEP induced by vonoprazan and the negative diagnostic results, we present this case to explore diagnostic limitations.

## Case presentation

**Figure 1:**
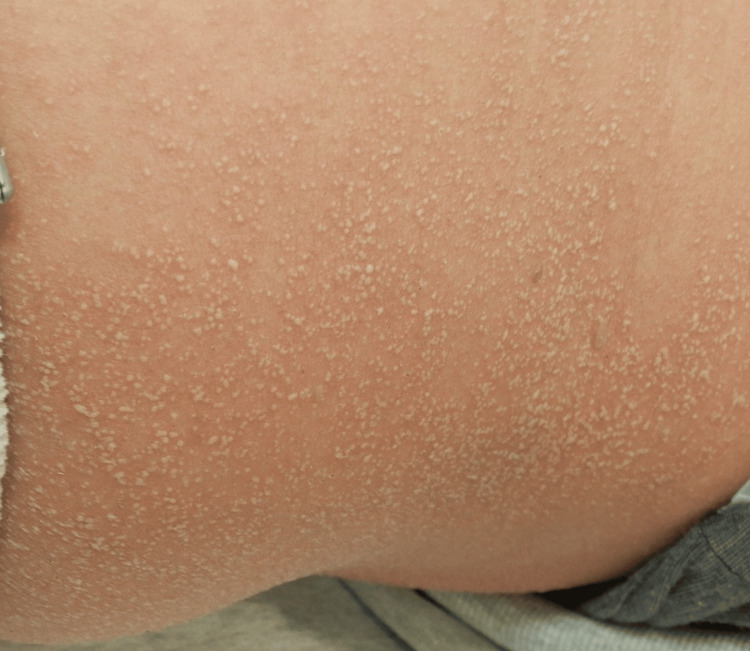
The entire skin surface appeared erythematous with diffused numerous small pustules, indicating AGEP (1-2 mm size). AGEP: acute generalized exanthematous pustulosis.

A 66-year-old woman with a past medical history of hypertension and ulcerative colitis had been taking mesalazine, olmesartan, and famotidine for over five years. Six months prior, she was diagnosed with left-sided breast cancer and underwent surgery followed by adjuvant chemotherapy with paclitaxel. The first and second infusions of paclitaxel were well tolerated, without any adverse events, including dermatological manifestations. At the time of the third paclitaxel infusion, due to worsening anemia and gastrointestinal symptoms, iron preparation and vonoprazan were added on the same day. Two days after administration of paclitaxel (210 mg/m²/3 weeks), iron preparation, and vonoprazan (20 mg/day), she developed widespread erythema, which progressed further the following day. She presented to our dermatology department with a generalized erythroderma involving the trunk and extremities, scattered with small pustules (Figure 1).

The clinical findings were inconsistent with DIHS/DRESS. Vital signs revealed a high fever of 39.6°C, although her blood pressure, oxygen saturation, and level of consciousness were preserved. She had pruritus and burning sensation. Laboratory investigations showed marked leukocytosis (30,100/μL; reference: 3,500-9,000/μL) and elevated CRP (2.3 mg/dL; reference: 0-0.3 mg/dL), indicating acute inflammation. Eosinophil counts were within the normal range, and there was no evidence of hepatic or renal dysfunction. The laboratory findings are consistent with AGEP. Peripheral blood smear showed no atypical lymphocytes. Serologic tests for herpes simplex virus (HSV), cytomegalovirus (CMV), and Epstein-Barr virus (EBV) IgM antibodies were all negative. All laboratory examination data is shown in Table 1.

**Table 1:** The result of laboratory examination. RR: reference range; AST: aspartate aminotransferase; ALT: alanine aminotransferase; LDH: lactate dehydrogenase; CRP: C-reactive protein; BUN: blood urea nitrogen, CMV: cytomegalovirus; EB: Epstein-Barr; HSV: herpes simplex virus; VCA: viral capsid antigen.

Variable	Patient value	RR, adults
AST	27 U/L	11-33 U/L
ALT	33 U/L	6-37 U/L
Albumin	3.4 g/dL	3.8-5.0 g/dL
Total bilirubin	0.6 mg/dL	0.2-1.2 mg/dL
LDH	432 U/L	135-214 U/L
CRP	2.9 mg/dL	< 0.50 mg/dL
BUN	8.2 mg/dL	8-20 mg/dL
Creatinine	0.63 mg/dL	0.6-1.0 mg/dL
WBC	30,100/μL	3,500-9,000/μL
Hb	8.9 g/dL	12-16 g/dL
CMV IgM	Nonreactive	Nonreactive
CMV IgG	Nonreactive	Nonreactive
EB VCA-IgM	Nonreactive	Nonreactive
EB VCA-IgG	Reactive	Nonreactive
HSV IgM	Nonreactive	Nonreactive
HSV IgG	Reactive	Nonreactive
Abnormal lymphocyte	0%	0%-5%

**Figure 2:**
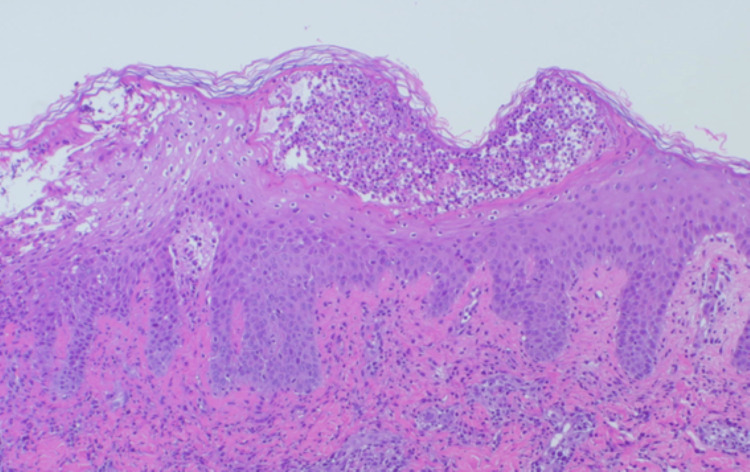
Subcorneal pustules were observed, containing infiltrating neutrophils. The epidermis exhibited spongiosis, and there was marked inflammatory cell infiltration around the dermal capillaries (hematoxylin and eosin (HE) staining, ×100).

**Figure 3:**
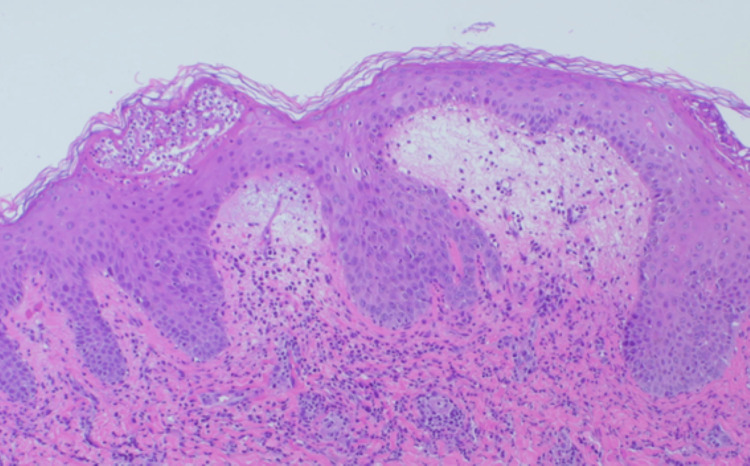
In addition to subcorneal pustules, edema in the upper dermis and partial infiltration of inflammatory cells into the basal layer of the epidermis were observed (hematoxylin and eosin (HE) staining x100).

Histopathological examination of a skin biopsy revealed subcorneal pustules (Figure 2), liquefaction degeneration, lymphocytic infiltration into the basal layer, and inflammatory infiltrates surrounding superficial dermal vessels (Figure 3).

**Figure 4:**
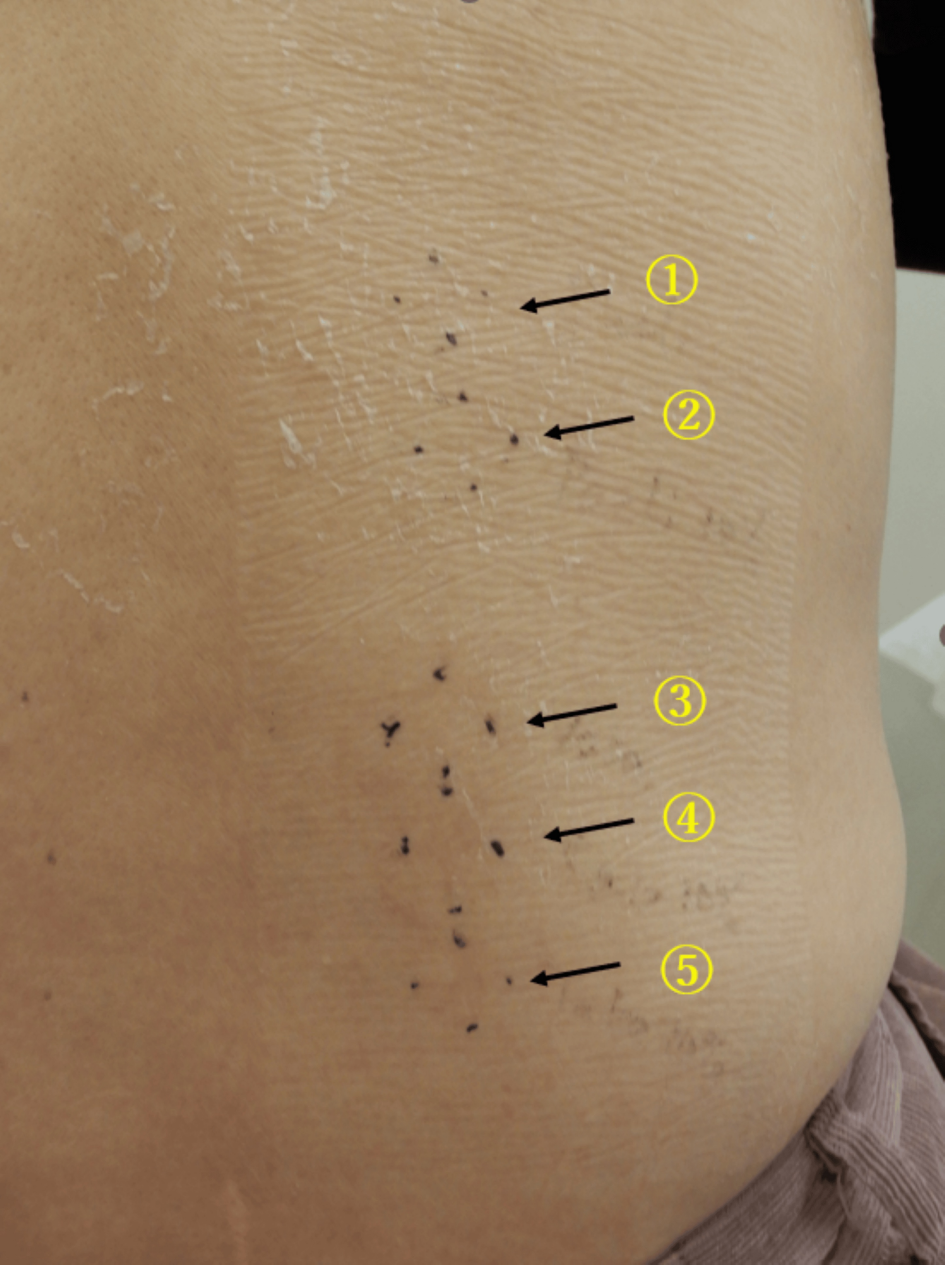
The result of patch test. 1: Paclitaxel diluted as if, 2: paclitaxel diluted with normal saline to a 10% concentration, 3: white petrolatum (as a control), 4: iron preparation diluted with white petrolatum to a 10% concentration, and 5: vonoprazan diluted with white petrolatum to a 10% concentration. No erythema, edema, or vesiculation was noted at any patch site, indicating negative test results.

Based on a EuroSCAR score of 12 (full score), a diagnosis of AGEP was established and other differential diagnoses were ruled out. Given the typical onset within days following drug administration and the exclusion of viral infections, paclitaxel, iron preparation, and vonoprazan were considered as possible causative agents. All three suspect drugs were discontinued, and treatment was initiated with topical clobetasol propionate and oral prednisolone at 20 mg/day. The skin lesions showed significant improvement within three days, consistent with the typical clinical course of AGEP. Since the skin symptoms remarkably improved after prednisolone administration, oral prednisolone was tapered rapidly every three days (15 mg → 10 mg → 5 mg → 2.5 mg) and discontinued after two weeks. Topical corticosteroids were also stopped after approximately two weeks, as the erythema had largely resolved, leaving only post-inflammatory hyperpigmentation. Patch testing with all three suspect agents yielded negative results (Figure 4).

**Figure 5:**
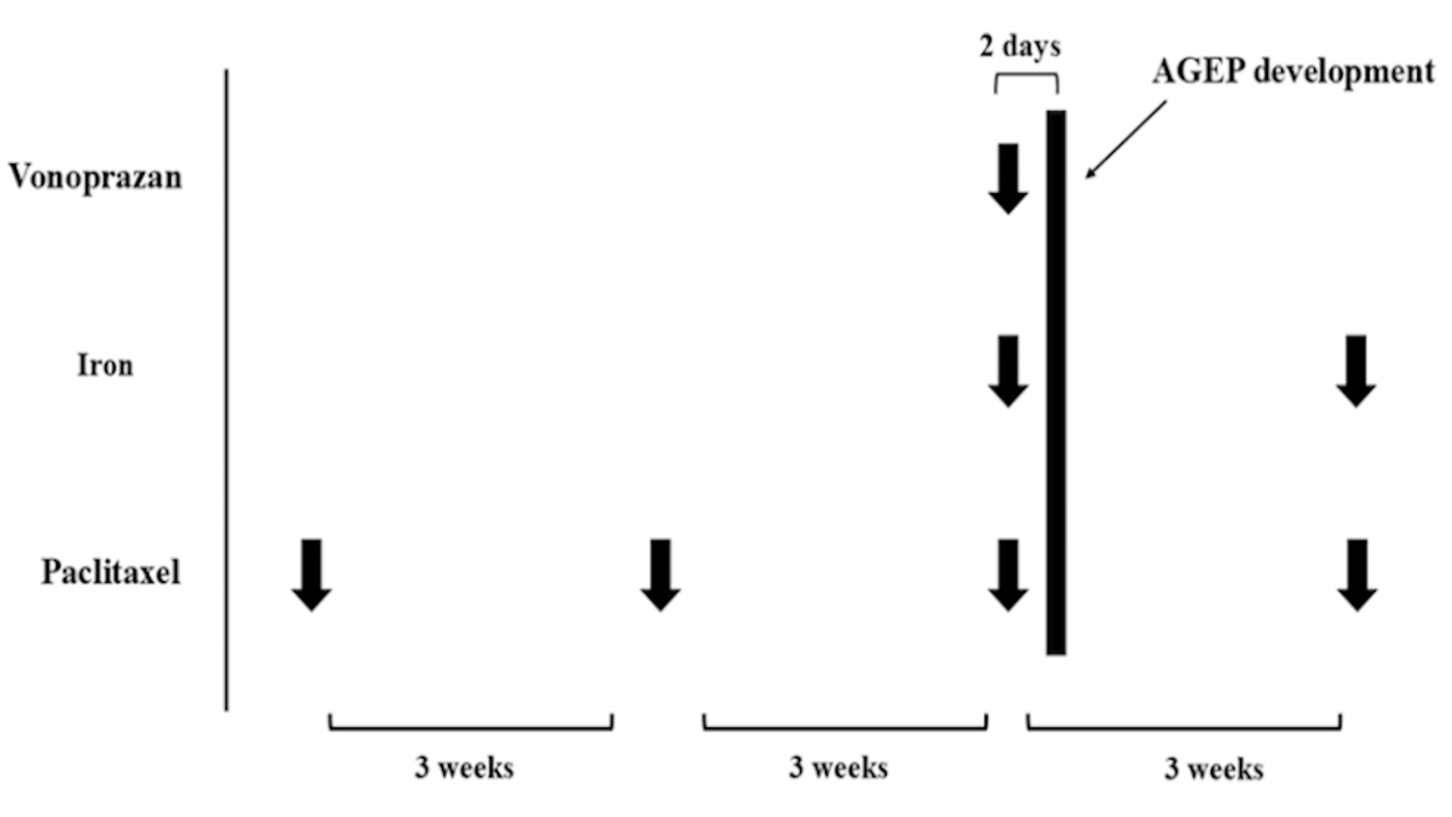
The timeline of drug induction. AGEP: acute generalized exanthematous pustulosis.

To our knowledge, AGEP induced by either iron supplementation or paclitaxel has not been previously reported. On the other hand, vonoprazan, like other proton pump inhibitors (PPIs), has been associated with severe cutaneous drug eruptions such as erythroderma, and PPIs are known risk factors for AGEP [12,13]. Therefore, we concluded that vonoprazan was the most likely causative agent in this case. As the patient wished to continue anticancer treatment and anemia management, we cautiously resumed paclitaxel infusion and iron administration under short-term corticosteroid prophylaxis three weeks after AGEP occurrence (prednisolone 25 mg/day for three days). No recurrence of AGEP was observed after steroid withdrawal, supporting the conclusion that neither paclitaxel nor iron preparation was responsible. The DLST for vonoprazan was performed but returned negative (stimulation index: 161%; normal range: 0-179). Given the potential risk of AGEP recurrence, intradermal testing, prick testing, and oral challenge with vonoprazan were not performed, in agreement with the patient’s preference. The timeline is shown in Figure 5. The patient is currently being managed with an alternative gastrointestinal agent and has remained free of AGEP recurrence.

## Discussion

This case is notable for its acute onset, widespread pustular rash, fever, and leukocytosis developing two days after vonoprazan initiation. Patch testing was performed for all three suspected drugs, and the results were negative. Previous reports have suggested that vonoprazan, similar to other proton pump inhibitors (PPIs), carries a risk of inducing generalized drug eruptions, including erythroderma [12,13]. In this case, the patient strongly wished to continue chemotherapy for breast cancer. After thoroughly discussing the potential risk of AGEP recurrence, paclitaxel and iron preparation were cautiously reintroduced, without any recurrence of skin symptoms. Based on this clinical course, vonoprazan was considered the most likely causative agent. To establish a definitive diagnosis, it is necessary to carefully evaluate each potential trigger. Although the viral tests conducted in this case were negative, various other factors, such as undetected viral infections or psychological stress, could also serve as triggers. Therefore, we acknowledge as a limitation that we were unable to completely rule out causes other than vonoprazan. Evidence regarding the sensitivity of patch testing in AGEP remains limited, with reported positivity rates varying widely from 18% to 75% [14,15]. This suggests that false-negative results may be more common than generally expected. Additionally, there have been reports of patch testing itself triggering AGEP skin lesions, indicating the need for caution when performing the test [8]. Currently, there are no standardized protocols regarding the optimal concentration or timing of patch testing in AGEP, highlighting the need for further investigation [13]. DLST, which was also performed in this case, is a commonly used in vitro assay for identifying causative agents in drug-induced hypersensitivity reactions [11]. It detects the activation and proliferation of drug-specific memory T cells and is generally considered a safe and non-invasive allergy test [16]. However, to date, no large-scale studies have assessed its sensitivity or specificity specifically for AGEP. While DLST can be helpful in identifying causative agents, false-negative results are not uncommon [11,17]. Moreover, discrepancies between patch testing and DLST results can occur, and ultimately, clinical judgment based on the overall course remains essential [10]. There is no single correct approach to how precisely drug eruption should be diagnosed and managed; decisions should be made through shared decision-making with the patient. Development of more sensitive in vitro diagnostic tools could improve diagnostic accuracy in AGEP.

In the present case, paclitaxel and iron preparation were considered critical for the patient's postoperative adjuvant therapy and management of chemotherapy-induced anemia. After discussing the risk-benefit balance with the patient, these agents were re-administered under concomitant oral prednisolone, without recurrence of AGEP. This clinical course further supported the diagnosis of vonoprazan-induced AGEP and suggested that the negative patch test and DLST results were likely false negatives. Although intradermal testing and prick testing were considered as additional diagnostic options, they were not performed due to the absence of AGEP recurrence and the patient's wish to continue chemotherapy without further invasive testing [11]. Oral challenge testing was also not performed, as it is generally contraindicated in severe drug hypersensitivity reactions due to the high risk of relapse and should only be considered when the benefits outweigh the risks [11]. Given the importance of paclitaxel in postoperative chemotherapy and iron supplementation in managing anemia, and considering that neither drug has been strongly associated with AGEP in prior literature, the decision to reintroduce them was based on a thorough risk-benefit analysis. The absence of recurrence following re-administration clinically confirmed vonoprazan as the likely causative drug.

This case highlights the importance of considering the limitations of diagnostic tests such as patch testing and DLST when evaluating AGEP. In cases where AGEP diagnosis is uncertain and essential drugs are involved, clinical judgment should override negative test results if patient safety permits. In instances where the causative agent cannot be clearly identified through these tests, clinicians must carefully assess the timing of drug exposure, known incidence rates of drug eruptions, and clinical context. For medications essential to patient prognosis, such as chemotherapy agents, it is crucial to weigh the therapeutic benefit against the risk of drug eruption recurrence and make decisions in close consultation with the patient. Further research is warranted to establish standardized protocols for patch testing in AGEP, including optimal concentrations and timing, as well as to improve the diagnostic accuracy of DLST.

## Conclusions

Acute generalized exanthematous pustulosis (AGEP) is an acute-onset dermatological condition characterized by edematous erythema, predominantly affecting intertriginous areas and the face, accompanied by the appearance of sterile, non-follicular pustules. We report a case of AGEP that developed two days after the administration of paclitaxel, iron supplements, and vonoprazan. Patch testing for all three agents yielded negative results; however, upon re-administration of paclitaxel and iron, no recurrence of skin symptoms was observed. Based on this clinical course, vonoprazan was identified as the most likely causative agent. As both patch testing and drug-induced lymphocyte stimulation testing have the potential for false-negative results, it is essential to recognize their limitations in the diagnosis of AGEP. Clinical decisions regarding drug re-administration should be made with careful consideration of the individual drug's clinical context, therapeutic necessity, and overall benefit-risk balance.
